# mHealth Solutions for Mental Health Screening and Diagnosis: A Review of App User Perspectives Using Sentiment and Thematic Analysis

**DOI:** 10.3389/fpsyt.2022.857304

**Published:** 2022-04-27

**Authors:** Erin Lucy Funnell, Benedetta Spadaro, Nayra Martin-Key, Tim Metcalfe, Sabine Bahn

**Affiliations:** ^1^Cambridge Centre for Neuropsychiatric Research, Department of Chemical Engineering and Biotechnology, University of Cambridge, Cambridge, United Kingdom; ^2^Independent Researcher, Cambridge, United Kingdom

**Keywords:** app users, app reviews, digital health, mental health, mHealth, sentiment analysis, thematic analysis

## Abstract

Mental health screening and diagnostic apps can provide an opportunity to reduce strain on mental health services, improve patient well-being, and increase access for underrepresented groups. Despite promise of their acceptability, many mental health apps on the market suffer from high dropout due to a multitude of issues. Understanding user opinions of currently available mental health apps beyond star ratings can provide knowledge which can inform the development of future mental health apps. This study aimed to conduct a review of current apps which offer screening and/or aid diagnosis of mental health conditions on the Apple app store (iOS), Google Play app store (Android), and using the m-health Index and Navigation Database (MIND). In addition, the study aimed to evaluate user experiences of the apps, identify common app features and determine which features are associated with app use discontinuation. The Apple app store, Google Play app store, and MIND were searched. User reviews and associated metadata were then extracted to perform a sentiment and thematic analysis. The final sample included 92 apps. 45.65% (*n* = 42) of these apps only screened for or diagnosed a single mental health condition and the most commonly assessed mental health condition was depression (38.04%, *n* = 35). 73.91% (*n* = 68) of the apps offered additional in-app features to the mental health assessment (e.g., mood tracking). The average user rating for the included apps was 3.70 (SD = 1.63) and just under two-thirds had a rating of four stars or above (65.09%, *n* = 442). Sentiment analysis revealed that 65.24%, *n* = 441 of the reviews had a positive sentiment. Ten themes were identified in the thematic analysis, with the most frequently occurring being performance (41.32%, *n* = 231) and functionality (39.18%, *n* = 219). In reviews which commented on app use discontinuation, functionality and accessibility in combination were the most frequent barriers to sustained app use (25.33%, *n* = 19). Despite the majority of user reviews demonstrating a positive sentiment, there are several areas of improvement to be addressed. User reviews can reveal ways to increase performance and functionality. App user reviews are a valuable resource for the development and future improvements of apps designed for mental health diagnosis and screening.

## Introduction

The prevalence of mental health concerns and disorders has increased following the COVID-19 pandemic (1–6). Despite this, during the initial lockdown in the United Kingdom contact with mental health services fell (6). Therefore, an increase in the demand for mental healthcare is predicted (6). The increased demand on mental healthcare services alongside workforce shortages (7) pose major obstacles to timely and effective mental healthcare provision (8, 9). This is concerning as long wait times in mental health are associated with poorer outcomes including increased suicidal risk, poorer social adjustment, decreased treatment responses and a higher risk of comorbidities (10–17). In contrast, early intervention and at-home treatment for mental health can minimize hospital admissions, shorten hospital stays, and result in cost savings for healthcare providers (18–21). Therefore, finding faster ways to assess, triage and treat mental health patients is vital. Mental health screening can provide a fast way to identify patients who could benefit from additional, more comprehensive mental health assessments (22). Thus, screening could also identify patients whose mental health could be managed with self-help strategies and do not require formal treatment (23). Screening could additionally reduce strain on primary care services (24) which is vital considering that, as of 2018, General Practitioners (GPs) report that 40% of appointments are related to mental health concerns (22, 25). This could be accomplished *via* signposting to other services following mental health screening, as a case study conducted in a group of GP surgeries demonstrated that active signposting frees up 80 additional appointments per surgery each week (26). Additionally, screening could minimize the risk of overlooking the presence of a mental health condition, which could delay access to treatment and worsen their prognosis (27).

In this regard, mobile health (mHealth) tools, such as applications (apps), could reduce strain on and increase access to mental health support or services. Additionally, they can facilitate early identification of mental health disorders and support self-management (28). mHealth tools are convenient, instant, and scalable (24, 29), as well as empowering individuals in managing their mental health (28–30), without the restrictions imposed by traditional mental health services (i.e., lack of access and long waiting times) (28, 29). Apps can also aid in engaging typically hard-to-reach patient populations by reducing stigma and increasing help-seeking behaviors (30). This is increasingly important following the recent COVID-19 pandemic, during which already present health disparities have only widened (31). mHealth for screening and management of mental health issues have gained popularity in recent years (32). The current estimates for the number of mental health apps available for public use stands at between 10,000 and 20,000 (33). In addition, the recent COVID-19 pandemic has further highlighted the benefits of mHealth apps for mental health by offering patients the opportunity to access a variety of mental health support during the COVID-19 pandemic, when traditional face-to-face care was not possible (34).

Furthermore, several studies have demonstrated the acceptability of digital technologies for improving the accessibility of mental health care and screening (35, 36). In a recent cross-sectional study involving over 8,000 users of a depression-screening app, it was found that a high percentage (73.90%) of app users completed the screening questionnaire (37). A second, multinational study of a depression screening app showed that, of those who downloaded the app, over two thirds completed a depression screening (38). These findings indicate that questionnaires completed *via* apps are a potentially feasible method of delivery for mental health assessments.

However, there is evidence for low user engagement (39), and a high drop-out rate for mental health apps (40, 41), with the drop-out rate appearing to be higher in real-world settings compared to clinical trials (42). One possible explanation for high drop-out could be issues related to usability, which are widely recognized in mental health apps (43, 44). In a systematic review of engagement in digital mental health interventions, issues with usability are a substantial barrier (45). ORCHA, a digital health compliance reviewer, found that 29.6% of the reviewed mental health apps do not meet their quality thresholds (46). These quality thresholds include usability issues. In addition to lack of compliance with clinical standards and data protection guidelines (46). Additionally, other factors are put forward as being related to low app engagement including concerns about the security of user data (44, 45), the app not adequately meeting the users' needs, and the app being considered untrustworthy by users (44).

Publicly available app reviews and ratings can provide a wealth of information regarding user perspectives and usability issues (47–55). Indeed, ratings are a key decision-making tool for whether a user downloads and uses an app (49) and reviews can highlight key issues which may not be reflected in ratings alone (50). In studies analyzing user reviews of mental health apps including cognitive behavioral therapy (CBT) and mood monitoring apps, main findings emphasize usability and visual appeal (43, 44, 51–55). In addition, complaints reported by users seem to center on poor design, bugs, and issues related to content with a lack of clear information on how to use the app. In extreme cases, some users report that usability issues caused them to immediately stop using the app (43).

Insights from app user reviews of general mental health apps identified a mental health assessment as a feature users perceived as positive (55). Despite this however, to our knowledge, no studies to date have focused on user perceptions of screening and/or diagnostic assessments included within apps designed for mental health. Considering how essential early screening is, both for patient outcomes (10–21) and potentially for reducing strain on care services, understanding user perception of mental health apps which offer a screening and/or diagnostic assessment is essential.

Therefore, we set out to conduct a review of publicly available apps which offer a self-administered mental health screening and/or diagnostic assessment. Additionally, we aimed to investigate the content of user reviews, with a focus on themes related to the mental health screening and/or diagnostic assessment offered within the app.

Sentiment analysis was employed to determine user perception of their experiences using the app. Furthermore, thematic analysis was used to identify both app feature themes related to the app in general and those specific to the included mental health screening and/or diagnostic assessment. Thematic analysis utilized additionally to identify which app feature themes were associated with discontinuation in app use.

Whilst there is an overall lack of consensus on how to analyze app reviews, a combination of sentiment and thematic analysis was the favored analysis method in previous studies (51, 54, 55). Sentiment analysis can be used to identify the feelings and attitudes expressed by an individual in relation to a specific area. It is a popular method of analysis for user reviews (56) as it can determine the overall opinion, either positive, negative, or neutral, within short, informal text passages. On the other hand, thematic analysis allows a rich investigation of themes and their frequency within the data (57), thereby aiding our understanding of key app features as reported by users. The methods and findings from this study can inform future research efforts in mining large app review datasets as well as informing a user-centric design of future mental health apps which offer a screening or diagnostic assessment.

## Methods

Due to the focus of the current study being on user perspectives of publicly available apps which offer a mental health assessment, searches of app stores were conducted. However, app stores are not designed for rigorous, reproducible searches (58). In an effort to address this, the current study was inspired by a scoping review approach to improve the transparency and reproducibility. The preferred reporting items for systematic reviews and meta-analyses extension for scoping reviews (PRISMA-ScR) checklist was used to improve reporting of the methods implemented and searches which were conducted (see Supplementary Material 1) (59). The current study was also registered as a scoping review with the Open Science Framework [OSF; (60)].

The scope of the review included mental health apps whose intended user populations were adults (18+) who were searching for an app which included a self-administered screening for or diagnosis of common mental health disorders. Apps of interest offered a self-administered question- and answer-based digital screening or diagnostic tools.

### Search Strategy

The current study used two different app search strategies, performed in July 2021. A manual search of the Apple and Google Play app stores was conducted by the first author (EF). The app store searches were performed using the search terms: (1) “Mental health assessment”, (2) “Mental health test”, (3) “Mental health symptom checker” and (4) “Mental health check-up”. The number of results were capped at a maximum of 200 apps per search term to provide a comprehensive view of the current landscape of available apps, while still being feasible for manual analysis.

The decision was made to use broad search terms to capture the experience of app searchers who are seeking a general mental health assessment of common mental health disorders, whilst still identifying apps designed for assessment of specific disorders. This is in line with previous similar literature focused on analysis of user reviews of mental health apps, which also favored general search terms when performing app store searches (51, 54, 55).

Additionally, the M-health Index and Navigation Database (MIND) (61) was searched to identify apps of interest. MIND is a publicly available database of mental health apps which have been reviewed against the American Psychiatric Association's App Evaluation Model. The filter, “Assessments/Screening”, was applied to the application library to narrow the search to apps of interest to the current study. Any relevant apps identified during the search of MIND were then accessed *via* the Apple and/or Google Play app store.

### App Selection Criteria

The inclusion criteria (Table 1) were developed in consultation with a practicing psychiatrist (SB). After the searches were performed (EF), duplicate apps from different search terms were identified and removed (EF). Independent reviewers (EF/BS/NMK) then screened all the identified app's app store descriptions against the inclusion criteria (Table 1). Apps were labeled as “exclude,” “include,” or “maybe”. Any disagreements regarding the labeling were discussed among the reviewers until a consensus was reached. After discussion, any apps which were still labeled as “maybe” (*n* = 7) were downloaded and checked for suitability (EF/BS).

**Table 1:** Inclusion and exclusion criteria for apps in the current study.

	**Inclusion criteria**	**Exclusion criteria**
Accessibility of the app	Available for download through the official Apple app store or Google Play app store, without a referral. Either available for free or at cost, which may or may not offer in-app purchases	Not publicly available (i.e., requires a referral from a healthcare provider to access)
Intended population for the app	Intended for use in the general adult population (18+)	Intended for use in a specific population (i.e., pregnant individuals or individuals in the perinatal period, veterans/ active service members, refugees)
Assessment offered within the app	Any app which offers a self-administered, question-and-answer based mental health assessment (i.e., a questionnaire, conversational agent)	An app which does not offer a self-administered question-and answer-based mental health assessment (e.g., Rorschach test or designed to be administered by a healthcare professional)
Mental health condition assessed in the app	Offers screening and/or diagnostic assessment for any of the following conditions/symptoms: Bipolar disorder (BD), Major depressive disorder (MDD), Obsessive compulsive disorder (OCD), Generalized anxiety disorder (GAD), Agoraphobia, Social phobia, Panic disorder, Insomnia, Schizophrenia, Psychosis, Eating disorders (e.g., bulimia nervosa, anorexia nervosa), Personality disorders, Alcohol abuse, Substance abuse, Post-traumatic stress disorder (PTSD), Acute stress disorder, Adjustment disorder, Autism spectrum disorders (ASDs), Attention hyperactivity deficit disorder (ADHD), Self-harm, Suicidal thoughts and/or suicidality risk	Offer screening and/or diagnostic assessment for: neuropsychiatric disorders (e.g., dementias), any disorders that are due to clinically confirmed temporary or permanent dysfunction of the brain, physical health disorder or measure (e.g., a heart rate monitor) OR The app does not exclusively screen for or diagnose a mental health condition (i.e., also screens for physical health conditions)

If the same app was identified in both the app stores, both were screened using the app store description from each store for suitability against the inclusion criteria. If both apps from the different app stores were deemed relevant, both were included in the dataset. Both were included to provide a complete set of user reviews between both stores and to account for any between-store app differences.

### Data Analysis

#### Descriptive Information and App Features

Descriptive information was manually retrieved for each included app from the description provided in the app store. This included information about the cost and in-app purchases, additional app functions (i.e., if the app offers self-help advice or strategies), the number of mental health conditions screened in the app, which specific mental health condition(s) the app assessed and whether the app had a medical device certification. The apps identified using the MIND database were found in the relevant app stores, and descriptive data was collected from app store descriptions. All the descriptive app data was collated into an Excel spreadsheet (see Supplementary Material 2.1).

#### App Review Extraction

The app user review selection followed a method utilized by previous studies with a similar focus (48, 51). Reviews can be organized by using filters in both of the app stores included in the current study. Given the current study's focus on users' perspectives of mental health screening and/or diagnostics apps, it was decided to only analyze a subset of app reviews filtered by “most helpful” and by date. “Most helpful” reviews are determined within app stores by users up- or down-voting other users' reviews as either “Helpful” or “Not helpful”. Therefore, it is likely that the resulting sample includes reviews that users are interacting with and find most relevant. Additionally, a previous similar study also sorted reviews by “helpfulness” in an attempt to ensure there was a mix of both positive and negative reviews (54). However, differing from the previous study, the scope of included reviews was also limited to reviews submitted within the last 6 months. This was done in an effort to retrieve reviews which were likely relevant to the current app version.

In order to extract the “most helpful” user reviews and relevant metadata for these apps, scraping of the app stores was performed using open-source code (62) for the Google Play app store, and was performed in Node.js using the app-store-scraper module (63) for the Apple app store. The review extraction from the app stores was performed in August and September of 2021. If an app from the dataset was available for download on both the Google Play and Apple app stores then any relevant reviews from both app stores were scraped.

#### Sentiment Analysis

A sentiment analysis was manually conducted within an excel spreadsheet. The sentiment of each review was determined through consensus of at least two independent reviewers. Each review was manually labeled as either “positive”, “negative” or “neutral” (EF) depending on their sentiment. The reviews were manually re-analyzed under blinded conditions (BS/NMK). Any disagreements on the sentiment labeling of the reviews were discussed by all authors until a consensus was reached. Any reviews not written in English or reviews where a sentiment could not be determined (e.g., “Never really used this app much”.) were labeled as “unclear”. Any reviews which were not relevant to the focus of the study (i.e., questions to app developers, information about their mental health symptoms with no reference to the app, a review written on the behalf of someone else, a review of the clinician or service rather than the app itself) were labeled as “not relevant”. Any reviews labeled as “unclear” or “not relevant” were removed from the dataset.

#### Thematic Analysis

Following the sentiment analysis, any reviews lacking enough data to perform a thematic analysis (<5 words) were removed from the dataset. The thematic analysis was manually conducted in an excel spreadsheet following the Braun and Clarke framework (57).

The reviews were read and re-read until the first author (EF) was familiar with them and any initial ideas were noted. Initial codes were created (EF) and added to a coding framework with brief descriptions for each code. The reviews were then manually allocated codes under blinded conditions (EF/BS/NMK) using this coding framework. Any inconsistencies in the code allocations between the authors (EF/BS/NMK) were discussed until a consensus was reached. During the thematic analysis, every review included in the dataset received its final coding based on the consensus of at least two independent reviewers.

The identified codes were then grouped into broader themes, independently by two reviewers (EF/BS), which were then discussed with the third reviewer (NMK) until consensus was reached. Once the thematic labeling was finalized and code/theme frequencies had been calculated, theme co-occurrence was calculated in Excel (see Supplementary Materials 2.3–2.6). This included determining which themes were commonly identified in combination within the user reviews.

To identify app features which were associated with app use discontinuation, reviews which referred to app use discontinuation were labeled during the thematic analysis (EF/BS/NMK). User reviews were labeled as commenting on app use discontinuation if the user stated so either explicitly (i.e., the user stated they stopped using the app, deleted the app, uninstalled the app or found a different app to use) or if the user implied they would not use the app again (i.e., the user review describing the app as a waste of time or the user not being able to use the app at all).

Additionally, the thematic analysis was compared against the sentiment analysis in Excel. This analysis was performed in order to determine the context in which specific app features were mentioned in the review.

## Results

### Description of Included Apps and Review Extraction

**Figure 1:**
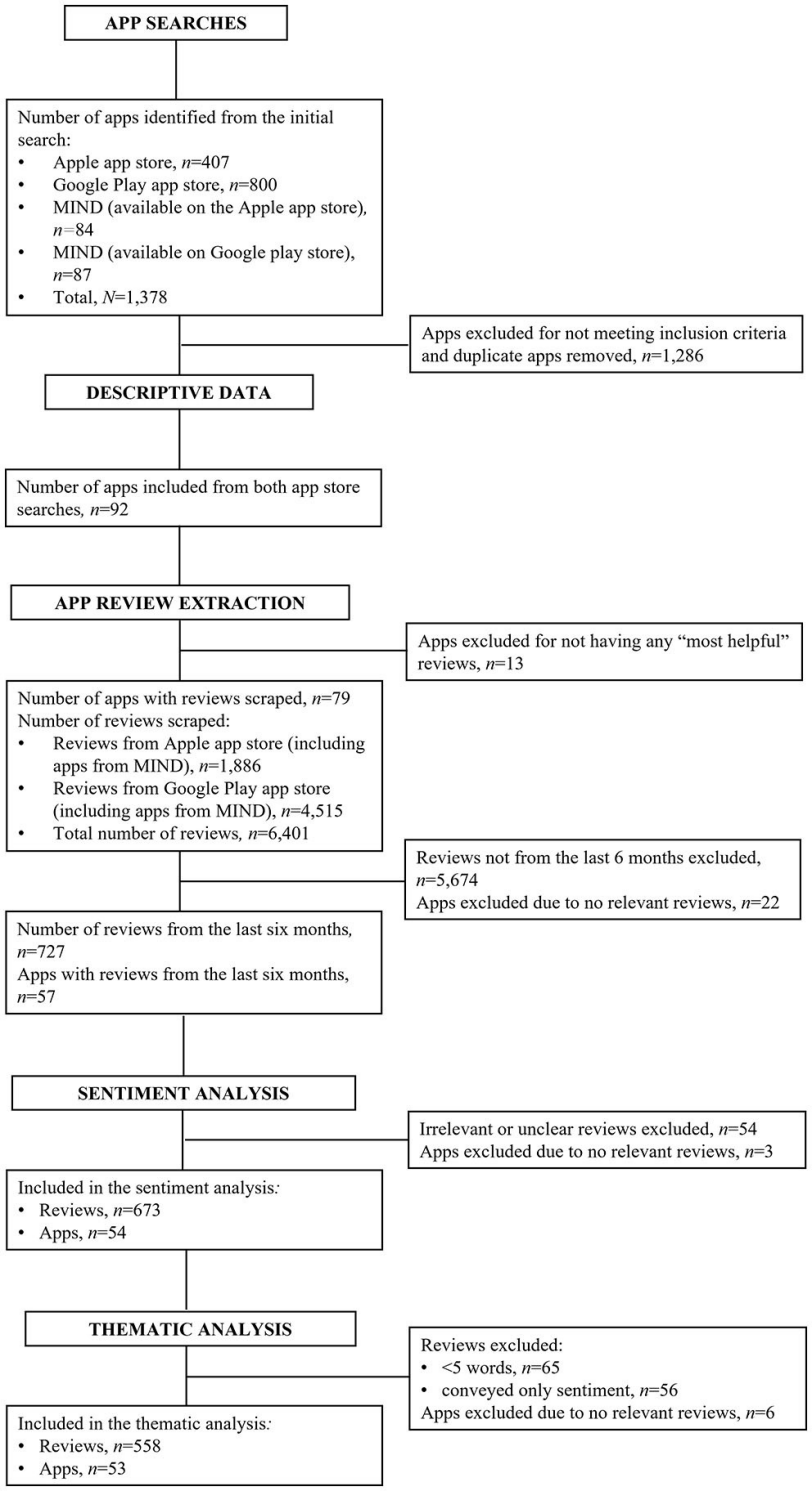
PRISMA flow chart of included apps and reviews. ***Key***. MIND, Mobile Health Index and Navigation Database.

The final app sample included 92 apps, comprising 69 apps from the Google Play app store and 23 apps from the Apple app store (Figure 1). Twelve of the identified apps (13.04%) were available for download on both the Google Play and Apple app stores.

**Figure 2:**
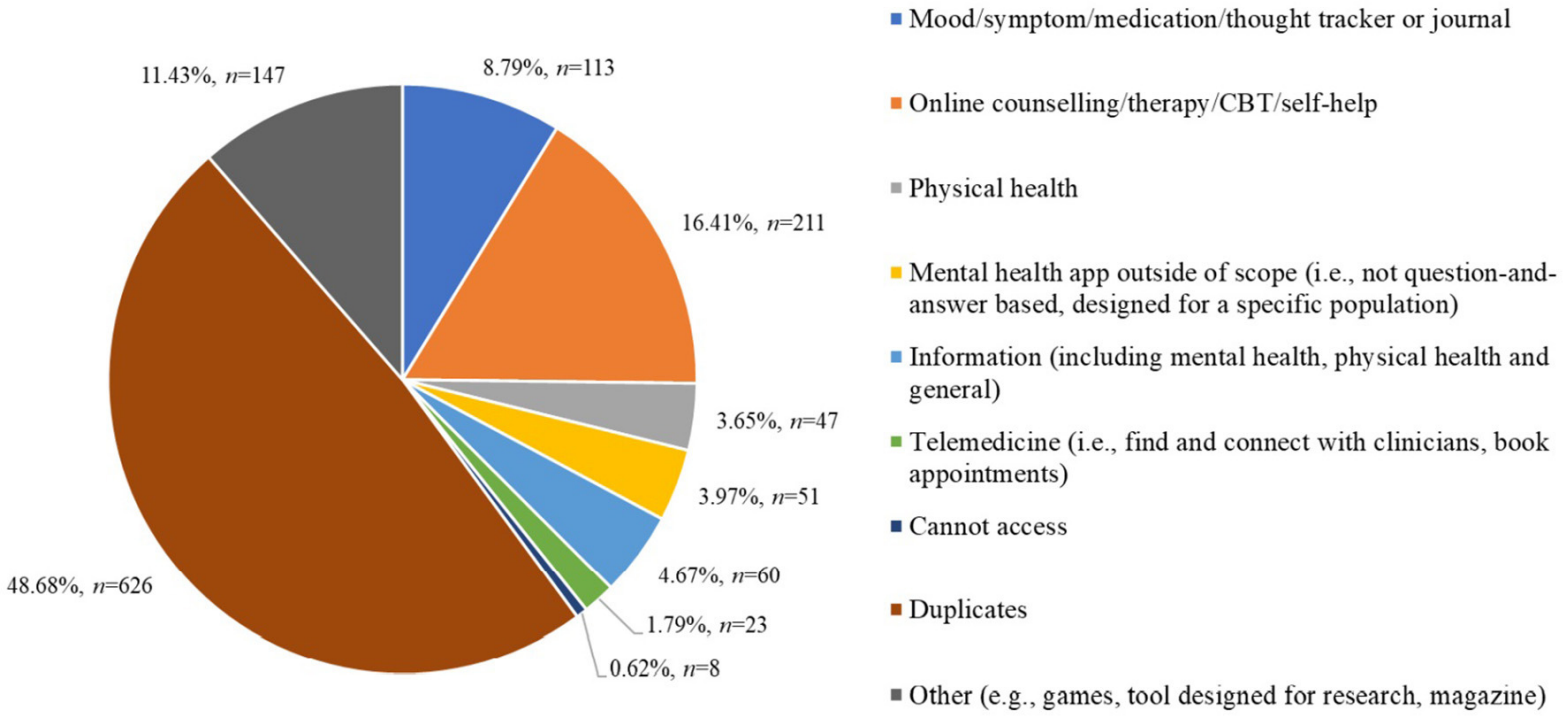
Number of apps excluded within a given exclusion category (*n* = 1,286). ***Key***. CBT, cognitive behavioral therapy.

Of the apps identified in the searches of the app stores (*N* = 1,378), 6.67% (*n* = 92) were relevant to the focus of the study, 1,286 apps were excluded as they did not meet the inclusion criteria of the study (Figure 2).

The majority of the included apps offered mental health screening (91.30%, *n* = 84) and only one of the included apps offered a diagnostic assessment. In a subset of the apps (7.61%, *n* = 7) it was unclear whether they offered mental health screening or diagnosis. Just under half of the included apps (46.74%, *n* = 43) offered a disclaimer in the app description (i.e., to consult a doctor after receiving their results, that the result is not a diagnosis, or only to use the results for educational purposes).

**Figure 3:**
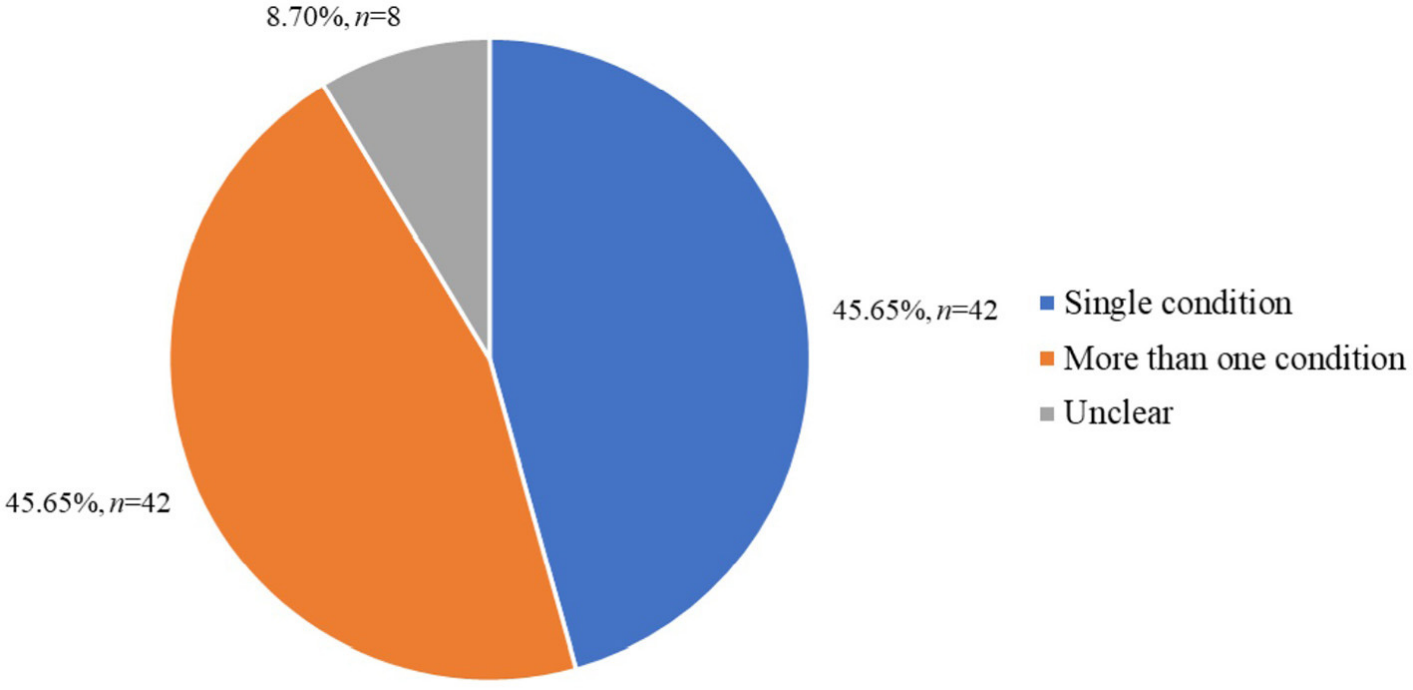
Apps by number of conditions assessed (*N* = 92).

Of the included apps, the same proportion of apps assessed a single mental health condition (e.g., depression) as compared to apps which assessed more than one mental health condition (Figure 3). The most commonly assessed mental health conditions within the included apps was depression (38.04%, *n* = 35), followed by anxiety and/or anxiety spectrum disorders (i.e., generalized anxiety disorder, social anxiety disorder, panic disorder; 30.43%, *n* = 28) and bipolar disorder (11.96%, *n* =1 1). Eight apps (8.70%) were unclear as to which conditions they assessed within their app store description. Of the apps which assessed more than one condition, 13 (30.95%) did not list all the conditions they assessed in their app store description.

All of the included apps were free to download, with 22.83% (*n* = 21) of these apps offering in-app purchases. The majority of the apps (73.91%, *n* = 68) offered additional features in addition to the mental health assessment (e.g., the ability to track changes in symptoms over time, self-help exercises, the ability to connect virtually with a clinician). The most common additional app feature identified using the app store descriptions was a tracking or journaling feature which allowed the user to save and monitor inputted data (i.e., mood, symptoms, thought patterns) over time (42.39%, *n* = 39 apps with tracking functionality). This was followed by information and/or psychoeducation (39.71%, *n* = 27) and self-help strategies (35.29%, *n* = 24). Twenty-four of the identified apps (35.29%) offered only a mental health assessment with no additional features. See Supplementary Material 2.1 for the full list of app features and information.

Once the app reviews were extracted, apps with no relevant reviews (i.e., the code did not scrape them from the app store or reviews which were not relevant once the filters for data selection were applied) were excluded (*n* = 35). Seven hundred and twenty-seven reviews were identified for inclusion from the remaining 57 apps. Of these reviews, 16 were categorized as “unclear” and 35 were categorized as “not relevant” and were thus removed from the dataset. Once the “unclear” and “not relevant” reviews were removed, 676 reviews were included in the sentiment and star rating analysis from 54 apps (Figure 1). Please see Supplementary Material 2.8 to see a distribution breakdown of the number of reviews per app.

### Sentiment Analysis and Star Ratings

**Figure 4:**
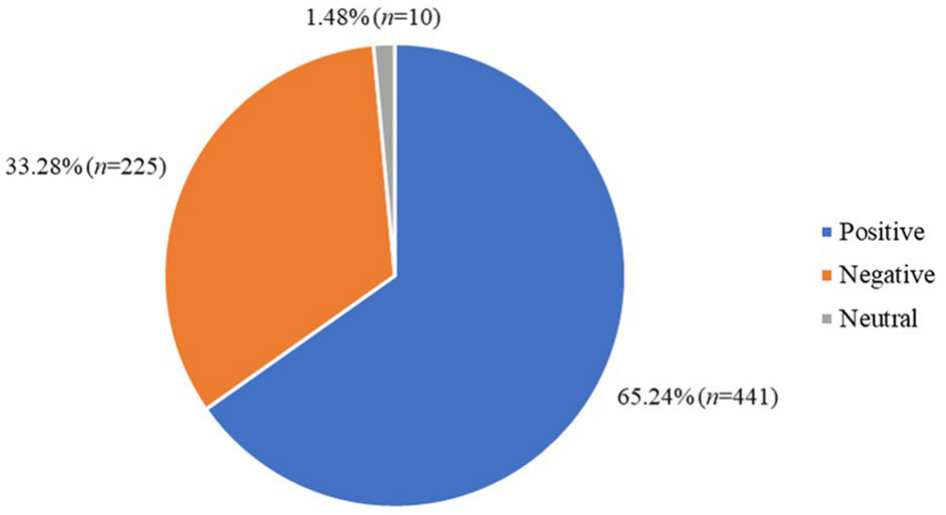
Frequencies of different sentiments of the reviews included in the sentiment analysis dataset (*n* = 676).

Just under two-thirds of the reviews had a positive sentiment (65.24%, *n* = 441) and just over a third of the reviews had a negative sentiment (33.28%, *n* = 225), the minority of the reviews had a neutral sentiment (1.48%, *n* = 10; Figure 4).

The average user star rating for the included apps was 3.70 (SD = 1.63) and just under two-thirds of the included apps had a rating of 4 stars or above from users (65.09%, *n* = 442).

A mismatch rate between the star rating and the sentiment analysis was calculated by considering a rating of 3 to be neutral, with a rating lower than 3 considered to be negative and a rating of above 3 to be positive. If the review and rating did not convey the same sentiment (i.e., a negative sentiment in the review but a rating of 4) then it was labeled as a mismatch. There was a mismatch between the review sentiment and the star ratings provided by the user in 9.02% (*n* = 61) of the included reviews. (For a full breakdown of the sentiment analysis, star ratings and mismatches see Supplementary Material 2.2).

### Thematic Analysis

Any reviews with <5 words from the sentiment analysis dataset were removed for thematic analysis (*n* = 59), leaving a dataset of 617 reviews. Of these, 58 reviews only conveyed sentiment and did not comment on a specific app feature or on app discontinuation. Therefore, only the remaining 559 user reviews, from 53 apps, had codes assigned to them.

**Figure 5:**
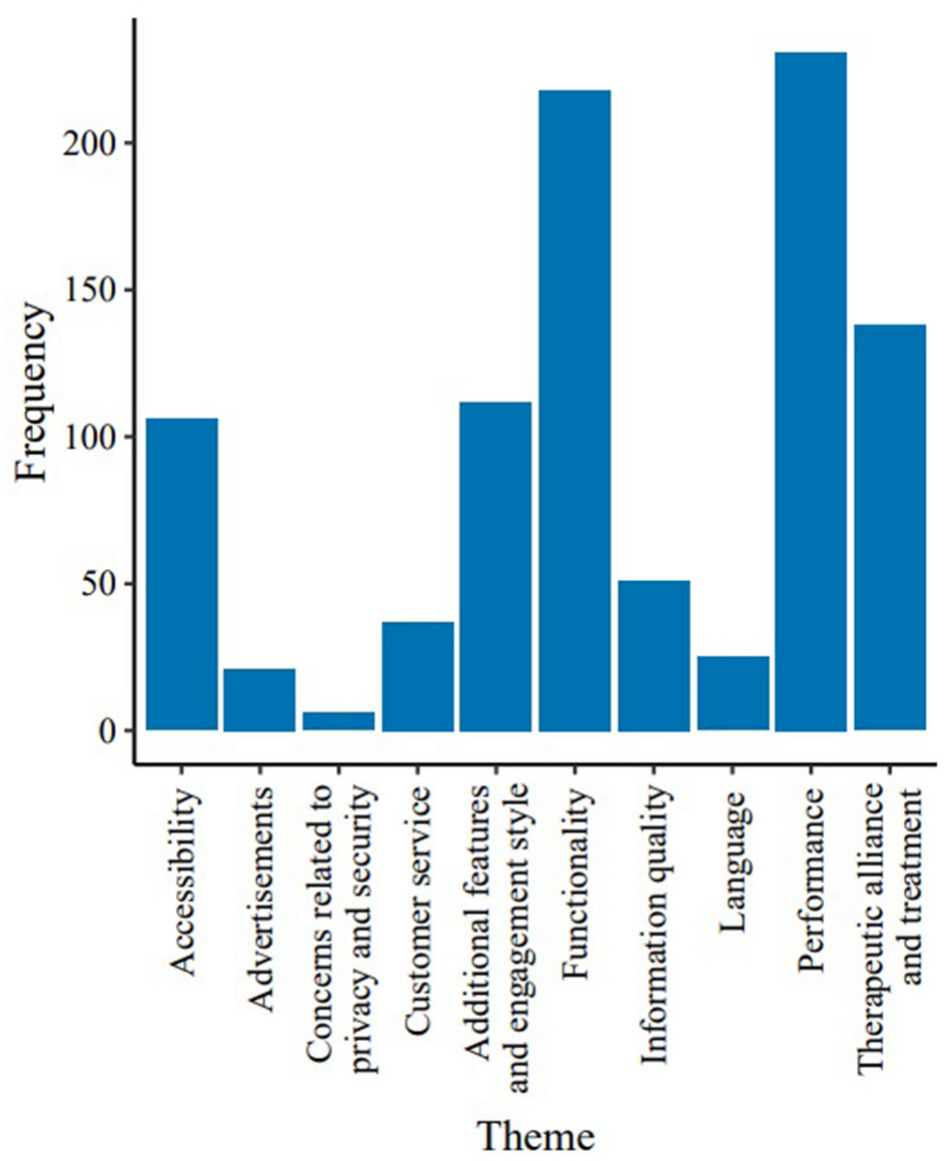
Frequencies of different themes identified in the thematic analysis (*n* = 558).

Ten themes were identified within the included user reviews (Table 2, Figure 5), comprising 64 individual codes (see Supplementary Material 2.3). 93.01% (*n* = 519) of the reviews were assigned to more than one theme.

**Table 2:** Frequency of the identified themes (*n* = 559; see Supplementary Material 2.3 for the breakdown of each theme into its codes and code frequencies).

**Theme**	**Description of the theme**	**Example review**	**Frequency, *n* (%)**
Performance	Refers to the quality of the screening and/or diagnostic assessment and results included within the app as well as the fit to app purpose (i.e., if the app is detrimental to mental health)	“*Awesome apI. You learn a lot about yourself through all of the tests they have available.”* [R134]	231 (41.40)
Functionality	Includes app features related to usability (i.e., ease of navigation), visual appeal, and technical issues	“*This update sucks. You have to search for the im feeling. When”m having a hard time that makes it worse. I do”t whos feedback was used but that was not a good idea.”* [R269]	219 (39.25)
Therapeutic alliance and treatment	Includes app features which offer the user the ability to connect with a clinician (i.e., find a local therapist, video call with a therapist) or a treatment intervention (i.e., self-administered cognitive behavioral therapy)	“*Amazing app lessons in CBT section worked to change my life.”* [R80]	140 (25.09)
Additional features and engagement style	Includes non-treatment-related additional in-app features other than the mental health screening and/or diagnostic assessment (i.e., tracking or logging of mood). Also includes features designed to increase engagement with the app (i.e., reminders to use the app)	“*This app is amazing it helps me to keep track of how”m feeling so her”s a 5 star review!!:-)”* [R165]	114 (20.43)
Accessibility	Related to the ease of accessing the app content including a paywall, cost, and inclusivity	“*Was perfect back when it was usable. Now every is behind a 100 buck a year paywall. Absolutely heartbreaking and has actively damaged my mental health progress.”* [R218]	103 (18.46)
Information quality	Either poor (i.e., in-accurate) or high (i.e., detailed) quality of the information provided within the app	“*Wonderful read and great info..So thankful to the author for writing this.”* [R87]	51 (9.14)
Customer service	Includes the quality of customer service provided by the app team and requests for additional app features within the app reviews	“*Unable to install application. It gives error. Tried callingriesterr but no response.”* [R98]	38 (6.81)
Language	Either poor (i.e., offensive) or high (i.e., professional) quality of language used within the app	“*Horrible app! So many misspellings, I could”t count them all! The“resul” of your tests make no sense, and you HAVE TO upgrade to get any information.”* [R222]	25 (4.48)
Advertisements (adverts)	The presence of advertisements (adverts) within the app	“*There are quite a few ads, but overall it was enjoying”* [R74]	21 (3.76)
Concerns related to privacy and security	User concerns related to the privacy/security or terms and conditions of the app (i.e., requests for unnecessary data)	“*Suspicious App Permission RequirementI. Why do you need access to my gallery? Why do you need to know my exact gps location?”* [R184]	6 (1.08)

In the quotations included in the thematic analysis, *R* and the following number refers to a number assigned to each review included in the thematic analysis. (See Supplementary Material 2.3 for the list of numbered reviews with their relevant codes). The review text included in the results was taken verbatim from the dataset so any spelling errors are as intended.

#### Performance

41.32% of the user reviews commented on app performance (*n* = 231). Half of the reviews which commented on the app performance mentioned that the app promoted mental health understanding and/or responsibility (50.65%, *n* = 117; “*Love that you can find so much out about yourself”* [R64]). The other most frequently observed indicators of performance in this theme were related to the perceived quality (i.e., good, or bad) of the assessment and/or questions included in the app (20.83%, *n* = 60; “*Simple to the point tests. Easy and they give rescources, which is nice”* [R105]) and the perceived accuracy of the app (10.07%, *n* = 29; “*Very few questions in the test, and they are poorly written, hence its very inaccurate.”* [R119]).

#### Functionality

Over a third of the user reviews (39.18%, *n* = 219) commented on the functionality of the app. Just over a third of reviews in the functionality theme (35.62%, *n* = 78) were related to a bad app update, such as an update which made the app harder to use or introduced a paywall (“*I LOVE Youper but please fix the lag and confusing, cluttered environment from the recent updates!”* [R133]). Other frequent aspects of the functionality theme included the quality of the visual design (36.53%, *n* = 80) and whether the ease of app use (30.14%, *n* = 66).

#### Therapeutic Alliance and Treatment

Just over a quarter of the user reviews commented on therapeutic alliance and treatment (25.04%, *n* = 140). The majority of these reviews (66.43%, *n* = 93), commented on treatment features included in the app (e.g., CBT, therapy, or a prescription service) with self-help being identified as the most frequently offered treatment from the reviews commenting on a treatment feature (51.43%, *n* = 72; “*What a great tool for improving mental health. I love doing my daily personalized brain trainings!”* [R527]). Just over a quarter of user reviews commenting on therapeutic alliance and treatment (27.54%, *n* = 38) referenced functionality which could connect a user with a clinician (e.g., GP, therapist, crisis hotline; “*It's a good way to link in with your Doctor. It's nerve racking to do on a personal aspect, but the app is easy and smooth to use.”* [R229]), with an additional 9.42% (*n* = 13) of reviews referencing functionality which gave users the ability to share their app data with their clinician(s) (“*Extremely helpful! Its great to be able to connect with my dietician and have the accountability piece. Its also really helpful to be able to get feedback so that I have more confidence in my ability to portion”* [R294]).

#### Additional Features and Engagement Style

20.39% (*n* = 114) of the user reviews referred to additional in-app features and engagement style offered in addition to the screening and/or diagnostic assessment. Tracking/logs/journaling was the most commonly identified feature within the theme of features and engagement style, present in 69.64% (*n* = 78; “*Helps me to keep track of my anxiety easily”* [R42]) of user reviews. Additional features designed to increase app engagement (e.g., rewards, motivational quotes or affirmations, reminders and/or app notifications) were present in 33.93% (*n* = 38) of reviews (“*[…] Every time I log a meal it gives me a coping skill or a positive statement or a cute cat picture or some other reward […]”* [R429]; “*[…] The reminders really help keep me on track.”* [R118]).

#### Accessibility

18.43% (*n* = 103) of the user reviews commented on the accessibility of the app. The most frequently identified app feature related to accessibility was a paywall 73.79% (*n* = 76; “*An app that used to be so useful, is literally garbage now. The purpose of app seems silly now that's only available behind a paywall, considering that most people from target audience cant afford/manage that. Very disappointed. Please request the creators to make the app free again. Even the older, simpler version would work for free users”* [R370]). Additionally, 24.27% (*n* = 25) of reviews related to accessibility commented on the app being non-accessible or non-inclusive (i.e., the app being overly expensive, the app not recognizing all gender identities and only acknowledging biological sex, the app design being unsuitable for individuals who are visually impaired or neurodivergent).

#### Information Quality

9.12% (*n* = 51) of reviews commented on information quality. Over three-quarters of reviews related to information quality were positive (76.47%, *n* = 39; “*This is an amazing app, very informative, covers a broad spectrum of mental health issues. Great format easy to use and easy to understand. Thank u”* [R210]). The majority of app reviews which commented on the app including poor information (*n* = 11; “*[…] Then when you actually start the program is literally a joke and the same info google could teach you.”* [R389]) stated that the information was too basic (72.73%, *n* = 8).

#### Customer Service

6.80% (*n* = 38) of the user reviews commented on the customer service offered by the app team. A request for an app feature (e.g., to simplify the app design, to allow the user to save their app data, allow more flexible tracking/logs/journaling) was the most frequently identified code included within the customer service theme (63.16%, *n* = 24; “*I wish I could create an account to save the data.”* [R72]). 26.32% (*n* = 10) of reviews identified as commenting on customer service mentioned the quality of a response from the app development team (e.g., either helpful: “*[…] after I got charged prior to payment, it got fixed and I'm thankful they were able to quickly get the situation sorted out and give me a refund.”* [R303]; or no response: “*Unable to install application. It gives error. Tried calling developer but no response.”* [R98]).

#### Language

4.47% (*n* = 25) of the user reviews commented on the quality of language used within the app. Over three quarters (76%, *n* = 19) of these reviews stated that the language used within the app was poor, most commonly due to poor translation or being written by a seemingly non-proficient English speaker (36.84, *n* = 7;“*very inarticulate, as though the dev doesn't speak English”* [R44]). In addition, 21.05% (*n* = 4) of reviews commenting on poor in-app language stated that the app included offensive or inappropriate language (“*We we're insulted to read within the first paragraph of D.I.D [...]”* [R288]). The use of offensive or inappropriate language was only reported in reviews of apps designed for personality disorders and was more frequent in apps for dissociative identity disorder (DID; 75.00%, *n* = 3).

#### Advertisements

3.76% (*n* = 21) of the user reviews commented on advertisements included within the app. Of these, 71.43% (*n* = 15) stated that the app contained too many adverts (“*Too many adds per test. There were 4 in my test 1 banner ad. And 3 that took me out of the test. I may use again but over time this would cause me to yeet”* [R284]). In contrast, 28.57% (*n* = 6) of user reviews stated that the app had no or well-placed adverts (i.e., they are well-placed within the flow of the app or assessment and so are not overly distracting; “*Not a bad lil app here. Even with the ads BECAUSE and only because they are spaced almost perfectly in your tests.”* [R186]).

#### Concerns Related to Privacy and Security

1.07% (*n* = 6) of reviews indicated concerns related to privacy and security, including suspicious data requirements or terms of service (“*Can't opt out of an extremely nefarious privacy policy. No thanks.”* [R84]). The majority of the reviews which mentioned concerns about privacy and security (3.33%, *n* = 2) commented on “suspicious” requests to access phone data outside of the app which users perceived as unnecessary (e.g., access to the user's location or their photographs).

### Theme Co-occurrence

The most common theme co-occurrence was accessibility and functionality (4.84%, *n* = 27) (Table 3).

**Table 3:** Frequency of theme combinations with five or more instances in the dataset (*n* = 559; see Supplementary Materials 2.4–2.5 for the remaining theme co-occurrences).

**Theme combination**	**Frequency (*n*, %)**
Accessibility AND Functionality	27 (4.83)
Functionality AND Performance	25 (4.47)
Additional features and engagement style AND Performance	23 (4.11)
Therapeutic alliance and treatment AND Performance	19 (3.40)
Therapeutic alliance and treatment AND Functionality	13 (2.33)
Therapeutic alliance and treatment AND Additional features and engagement style AND Performance	12 (2.15)
Additional features and engagement style AND Functionality	11 (1.97)
Functionality AND Customer service	11 (1.97)
Information quality AND Performance	9 (1.61)
Language AND Performance	8 (1.43)
Information quality AND Functionality	7 (1.25)
Therapeutic alliance and treatment AND Additional features and engagement styles	7 (1.25)
Therapeutic alliance and treatment AND Functionality AND Performance	7 (1.25)
Accessibility AND Performance	6 (1.07)
Additional features and engagement style AND Functionality AND Therapeutic alliance and treatment	6 (1.07)
Therapeutic alliance and treatment AND Additional features and engagement style AND Performance	5 (0.89)

Several themes were more commonly identified in the review set in combination with another theme than on their own. The additional features and engagement style theme was more frequent in combination with the performance theme (4.12%, *n* = 23; “*Very good app. I love the quote of the day and the tests are fun and accurate”*. [R144]) than the additional features or engagement style theme alone (3.41% *n* = 19). The customer service theme was more frequent in combination with the functionality theme (1.97%, *n* = 11; “*I downloaded this app and I answered about your questions, Then I went to create an account and every time I press on create account it does nothing. So I'm not going to be able to save my progress. I sent feedback to the app developers with my technical issue but who knows when it will be resolved.”* [R565]) than the customer service theme alone (1.79%, *n* = 10). Finally, the language theme was more frequently identified in combination with the performance theme (1.43%, *n* = 8; “*The test may be good but the English version has many questions that need a more accurate translation. Some of the questions are impossible to understand.”* [R209]) than in isolation (0.72%, *n* = 4).

### Association Between Themes and Sentiment

Of the reviews included in the thematic analysis (*n* = 617), just under two-thirds had a positive sentiment (64.02%, *n* = 395). Just over a third of the reviews had a negative sentiment (34.85%, *n* = 215) and the remaining reviews had a neutral sentiment (1.13%, *n* = 7).

When comparing the review sentiment against themes (see Table 4), positive sentiment most frequently occurred in combination with performance (16.71%, *n* = 66). On the other hand, negative sentiment was more commonly identified in combination with the functionality theme (13.95%, *n* = 30). In addition, negative sentiment frequently occurred alongside the accessibility theme, in isolation (7.91%, *n* = 17), and in combination with both the functionality theme and app discontinuation (8.84%, *n* = 19; “*I prefer the old version, the new update is just bad and as someone who is broke and can't afford the subscription I can't even talk to the AI for free, I'm forced to go look for other applications.”* [R309]).

**Table 4:** Frequency of themes associated with either a positive or negative review sentiment with five or more instances in the data set (positive reviews, *n* = 395; negative reviews, *n* = 215; see Supplementary Material 2.7 for the remaining theme and sentiment co-occurrences).

**Sentiment**	**Theme**	**Frequency, *n* (%)**
Positive	Performance of the assessment	66 (16.71)
	Therapeutic alliance and treatment	35 (8.86)
	Functionality	26 (6.58)
	Additional features and engagement style **AND** performance	22 (5.57)
	Therapeutic alliance and treatment **AND** performance	18 (4.56)
	Additional features and engagement style	18 (4.56)
	Functionality **AND** performance	12 (3.04)
	Therapeutic alliance and treatment **AND** additional features and engagement style **AND** performance	12 (3.04)
	Therapeutic alliance and treatment **AND** functionality	10 (2.53)
	Information quality	9 (2.28)
	Customer service	9 (2.28)
	Additional features and engagement style **AND** functionality	8 (2.03)
	Therapeutic alliance and treatment **AND** additional features and engagement style	8 (2.03)
	Information quality **AND** performance	7 (1.77)
	Therapeutic alliance **AND** functionality **AND** performance	7 (1.77)
	Information quality **AND** functionality	5 (1.27)
	Additional features and engagement style **AND** functionality **AND** performance	5 (1.27)
Negative	Functionality	30 (13.95)
	Accessibility **AND** functionality **AND** app discontinuation	19 (8.84)
	Accessibility	17 (7.91)
	Performance of the assessment	11 (5.12)
	Functionality **AND** app discontinuation	11 (5.12)
	Accessibility **AND** app discontinuation	10 (4.65)
	Functionality **AND** performance	9 (4.19)
	Accessibility **AND** functionality	8 (3.72)
	Language **AND** performance	6 (2.79)
	Functionality **AND** customer service	5 (2.33)
	Accessibility **AND** performance	5 (2.33)

When considering themes in isolation, positive sentiment was more frequent across the identified themes than negative sentiment (Table 5). The highest proportion of positive sentiment compared to negative sentiment was identified in the therapeutic alliance and treatment theme (positive reviews: 86.43%, *n* = 121; negative reviews: 13.57%, *n* = 19).

**Table 5:** Sentiment associated with themes (see Supplementary Material 2.7 for the remaining theme and sentiment co-occurrences).

	**Number of reviews with**	**Number of reviews with**	**Number of reviews with**
**Theme**	**positive sentiment, *n* (*%*)**	**negative sentiment, *n* (*%*)**	**a neutral sentiment, *n* (*%*)**
Accessibility (*n* = 103)	24 (23.30)	78 (75.73)	1 (0.97)
Additional features and engagement style (*n* = 114)	98 (85.96)	14 (12.28)	2 (1.75)
Advertisements (adverts) (*n* = 21)	9 (42.86)	11 (52.38)	1 (4.76)
Concerns related to privacy and security (*n* = 6)	0	6 (100.00)	0
Customer service (*n* = 38)	20 (52.63)	17 (44.74)	1 (2.63)
Functionality (*n* = 219)	98 (44.75)	120 (54.79)	1 (0.46)
Information quality (*n* = 51)	39 (76.47)	12 (23.53)	0
Language (*n* = 25)	7 (28.00)	18 (72.00)	0
Performance (*n* = 231)	173 (74.89)	57 (24.68)	1 (0.43)
Therapeutic alliance and treatment (*n* = 140)	121 (86.43)	19 (13.57)	0

Negative sentiment was more frequent in 4 themes: functionality (negative reviews: 54.79%, *n* = 120; positive reviews: 44.75%, *n* = 98), accessibility (negative reviews: 75.73%, *n* = 78; positive reviews: 23.30%, *n* = 24), language (negative reviews: 72.00%, *n* = 18; positive reviews: 28.00%, *n* = 7), and advertisements (negative reviews: 52.38%, *n* = 11; positive reviews: 42.86%, *n* = 9), with the highest proportion of negative sentiment over positive sentiment identified in the accessibility and language themes (Table 5).

### Themes Associated With App Discontinuation

Seventy-five (13.42%) of the user reviews included in the thematic dataset (*n* = 559) were labeled as either explicitly (“*Really sad about the direction this app has taken since the last update :(Hope the developers actually listen to the hundreds of displeased customers. I'll be uninstalling”* [R233]) or implicitly commenting on app use discontinuation (i.e., the user uninstalling the app, stating that installing the app is worthless: “*Waste of tax payer money, all negative reviews on here are 100% true. DON'T WASTE YOUR TIME.”* [R135]; “*I have adhd and i took the test and it said i dont so it does not work DO NOT DOWNLOAD”* [R173]).

The most common themes associated with app use discontinuation were accessibility and functionality in combination (Table 6; 25.00%, *n* = 19; “*It started as an awesome app, now it gets worse with every update. The subscription price hit the ceiling and now they even hide the simple single-answer emotion tracking behind the paywall. Greedy owners trying to turn it into a cashcow. Uninstalling.”* [R314]). In reviews which were labeled as mentioning app use discontinuation, functionality, and accessibility, the most commonly identified codes were a paywall (52.63%, *n* = 10; “*[…] But now it's all just one big paywall that is impossible for me to use anymore. […]* [R424]) and a poor-quality update (42.11%, *n* = 8; “*[…] ever since you guys started with the new update(s), it's gotten so much worse. […]* [R445]).

**Table 6:** Frequency of themes associated with app use discontinuation with three or more instances in the data set (*n* = 75; see Supplementary Material 2.6 for the remaining theme co-occurrences).

**Theme(s) associated with app use discontinuation**	**Frequency (*n*, %)**
Accessibility **AND** Functionality	19 (25.33)
Functionality	11 (14.67)
Accessibility	11 (14.67)
Functionality **AND** Performance	4 (5.33)
Accessibility **AND** Functionality AND Performance	4 (5.33)
Functionality **AND** Customer service	3 (4.00)

## Discussion

The current study, inspired by a scoping review methodology, conducted searches of popular app stores to identify mental health apps which offered a screening and/or diagnostic assessment. Following these searches, we aimed to understand app user perceptions with a particular focus on the included assessment, *via* a qualitative analysis of the app's written reviews.

### Overview of App Landscape and Sentiment

The current study demonstrated that the majority of apps resulting from the store searches were not relevant. This finding illustrates the difficulties of identifying apps using app store searches from both a user and a research perspective, due to the nature of app stores which base results on factors beyond the search terms employed (64) and allow for search results to be influenced by App Store Optimization (65). Shen et al. (66) reported similar findings: when using the search term “depression” in app stores, over a quarter of the results are apps not related to depression. Additionally, they showed that a quarter of the excluded apps did not mention depression in either the app title or the app store description (66). The proportion of irrelevant apps was even higher in another study, finding that under a third of the apps identified in an app store search for apps related to depression were relevant to the condition (67).

Of the apps that were relevant to the inclusion criteria of this review, many lacked sufficient information for the user regarding the content of the app in the store description. Despite the majority of included apps only offering mental health screening, just under half of the included apps offered a disclaimer in their app description. This is potentially concerning, as without a disclaimer users of the app may consider the results of a screening assessment to be a formal mental health diagnosis and, thus may not consult with a clinician. In addition, whilst most apps reported the mental health conditions they assessed, just under 10% of the included apps were unclear as to which mental health disorders they assessed. Considering the overwhelming proportion of irrelevant apps identified in the initial search, the lack of clear information further contributes to the difficulties faced by users searching the app stores. Therefore, app developers should strive to provide more detailed and accurate information within app store descriptions. This would help users in both finding an app and in ensuring its suitability from the information provided.

With respect to the user perspectives, just under two-thirds of the included app reviews received star ratings of 4 stars or above. This indicates overall satisfaction with the apps currently available for mental health screening and/or diagnostic assessment based on star rating. Additionally, the majority of written reviews for mental health screening and/or diagnostic apps conveyed a positive sentiment. The sentiment analysis also revealed that a very small minority of reviews conveyed a neutral sentiment. This could be due to app users being more likely to leave a review when they have had a particularly good or poor experience (68; “*I don't usually review apps unless I am obsessed with them or absolutely hate them. […]”* [R464]). Therefore, whilst there seems to be an overall positive user experience reported within app reviews, some information on app features which users find acceptable but neither particularly positive or negative may be missed. Hence, app developers should explore further methods, aside from only ratings and reviews, such as in-app user surveys to capture a broader spectrum of user experiences (68). Previous research has demonstrated that asking users about their experiences directly increases the likelihood of collecting feedback (68), suggesting this is a viable option for collecting neutral feedback.

Whilst star rating and written review sentiment were both overall positive, the current study revealed that star ratings should not be considered in isolation. The rate of mismatch between rating and review sentiment reported in the current study, while low, shows that star ratings may not fully capture sentiment. In addition, by analyzing the written user reviews, app developers can uncover a wealth of insights beyond what app star ratings alone can provide. This is demonstrated in the results of the thematic analysis performed in the current study, which identified 10 distinct themes which are important to users. In fact, despite the majority of reviews having a positive sentiment and high star rating by engaging in qualitative analysis of the written reviews, negative feature themes (i.e., functionality and accessibility issues) were identified, which otherwise may not have been captured by these metrics alone. Additionally, almost all of the reviews included in the thematic analysis mentioned multiple themes, which indicates how much information is provided by the user in written reviews. Similar findings are reported in previous literature reports (53) indicating the complexity of features which users appreciate in a mental health app.

### Identified Themes

In line with previous literature of mental health app reviews, we identified similar themes including the quality of information provided and language used, the presence of advertisements and customer service amongst other larger themes. Additionally, we also identified themes not exclusively reported for mental health apps including functionality (69), accessibility (70), and concerns about privacy and security (69, 71–73). However, our analysis reveals theme frequencies and patterns which differ from previous findings.

In the current study performance was the most commonly mentioned theme by users suggesting that, when focusing on the screening and/or diagnostic assessment aspect of mental health apps, performance is considered an important feature by users. A commonly reported facet of the performance theme was a self-reported increase in the user's understanding of or responsibility for their mental health following completion of the mental health assessment. This finding builds on previous reports demonstrating how mhealth tools designed for mental health can be employed in order to empower individuals to self-manage their mental health (28–30, 74) or encourage a user to seek help from a healthcare professional (38). In addition to increasing the user's understanding and/or responsibility for their mental health, another facet of the performance theme was the quality of the diagnostic and/or screening assessment and the accuracy as perceived by the user. These dimensions of the performance theme are intertwined, as a high accuracy of the assessment is essential for ensuring that the insights the user is gaining about their mental health are correct and that any actions that are taken (i.e., self-help, seeking help from a healthcare professional) are appropriate for their specific needs. This is important to note as, while the current study only reported on self-perceived accuracy as determined by the users themselves, a recent systematic review and meta-analysis demonstrated that the accuracy of apps currently publicly available for mental health assessment is mixed, with some demonstrating poor discriminatory performance (75). Therefore, whilst the ability of apps which offer a mental health diagnostic and/or screening assessment to increase understanding and promote responsibility for mental health is considered an important feature by users, these insights may not be as accurate as users perceive them to be. Reassuringly, when investigating the relationship between sentiment and feature themes, positive sentiment is observed in just under three-quarters of the user reviews which comment on performance.

Whilst previous studies analyzing reviews of mental health apps instead found usability (44) to be the most common theme above themes such as accuracy, in the current study, the performance theme was closely followed by the functionality theme. Previous research has determined that usability issues (referred to as functionality issues in the current study) constitute the key weakness of mental health apps (44). Additionally, issues related to usability are the main fix request made by users (44). Within the current study, we found that the most commonly reported functionality theme aspect was related to quality of updates implemented. Many users reported that the update worsened the app by introducing functionality issues (i.e., becoming less visually appealing, harder to use and introducing in-app bugs) and accessibility issues, chiefly a paywall. Previous work shows that users appreciate regular updates to improve and update app content (44). However, similarly to the current study's findings, updates can also be a cause of frustration for users by introducing issues (43, 44). In fact, in a survey of 654 app users, just under a third expressed hesitation before updating an app, with just under half also reporting they had experienced issues with an app after updating it (76). Users reported issues related to app crashing, low app speed, changes to features included within the app, and bugs as the largest issues following an update (76). Additionally, the quality of visual design was found to be a major aspect of the functionality theme in the current study. The majority of these reported that apps were poorly designed, characterized by flaws such as the interface being too cluttered and overwhelming. Visual design has been identified as a key area of usability issues before (43), with users preferring a “clean” design (53). Within the current study, negative sentiment was frequently observed alongside the functionality theme. This finding supports previous literature findings which demonstrates that usability is most commonly mentioned in a negative context within user reviews (43, 44, 53, 55).

Previous studies had identified accessibility as the most frequent theme mentioned in user reviews (53). The current study instead identified accessibility being reported by users less frequently. In this dataset, I largest dimension of accessibility was the app having a paywall, perhaps because the majority of the apps included in this study were free with in-app purchases. A paywall seems to be a common theme across reviews of mental health apps, including CBT apps, in which users often requested increased access to free features within the app (77). Users of mood monitoring apps described, *via* reviews, feelings of frustration when they paid for an app which they then determined was unsuitable to their needs (53). This again, highlights the importance of including accurate information about the services provided and features included in the app, as well as which features the user will be expected to pay for.

Unlike previous work, the current study identified the theme of therapeutic alliance and treatment as a frequent theme within the user reviews. Particularly, many users commented on self-help aspects of the included apps (i.e., self-guided meditation, breathing exercises, coping skill programs). This is perhaps explained by self-help features being the most commonly offered treatment-related feature within apps. This finding demonstrates the ability of mental health apps to encourage self-management of some conditions (28). An additional dimension of the therapeutic alliance theme, is the ability to connect with and share data with clinicians. Users often reported that being able to share the data collected within their app with their clinician increased their clinician's insight into their condition. This attitude was also present in user reviews of CBT apps for depression (51), of apps designed for bipolar disorder (52), and mood monitoring apps (53).

In terms of the additional features and engagement style theme, a tracking/log/journaling function was the most commonly mentioned feature within user reviews. Recent research on people's use and perspectives on mood tracking support the view this app feature is generally perceived as useful (78) and positive (55) by users (78). Furthermore, tracking was the most commonly requested feature within apps designed for bipolar disorder (52). Additionally, tracking has been identified as a facilitator to user engagement in digital mental health intervention apps (45). Tracking can promote self-reflection (79), which was also observed in the current study. The results of the thematic analysis conducted in the current study revealed an overlap between the themes of additional features and performance. Specifically, the dimension of performance which was most frequently identified in combination with tracking/log/journaling, as this was increasing understanding and/or responsibility for mental health. Therefore, due to the positive perception of tracking, we recommend that mental health apps which include a screening and/or diagnostic assessment consider also adapting the assessment into a longitudinal tracking tool. The addition of this tool may help facilitate user engagement *via* long-term symptom monitoring (45), as well as further increasing the ability of an app to offer mental health understanding and/or responsibility. Additionally, a differing finding from previous studies is the frequency with which concerns about privacy or security were raised by users. Privacy is often mentioned as a foremost concern of mental health and non-mental health app users (44, 55, 71–73), but this was not reflected in our dataset. A study looking at mood monitoring apps intended for use in young people showed that the proportion of reviews commenting on privacy and security was 5.66% (53). Furthermore, user reviews of bipolar apps commented that concerns related to privacy and security were considered “dealbreakers” by users (52). By contrast, we report very few instances of users commenting on the privacy or security of the app within their reviews (1%). However, as mental health apps which include a screening and/or diagnostic assessment would capture potentially sensitive symptom and demographic data, any concerns related to privacy and security are worrying.

### App Discontinuation

Our findings suggest that reviews can also offer insights into reasons for app use discontinuation. With regard to our observations of app use discontinuation reported in the user reviews, this was higher than in previous reports (48). However, this difference could be due to our broader definition which also included implicit references to app use discontinuation. Considering that negative sentiment was commonly observed alongside functionality and accessibility, it is perhaps unsurprising that these themes were also key factors reported in app use discontinuation; in particular, a bad update and a paywall.

Complaints mentioning app use discontinuation related to poor-quality updates usually commented on the app becoming less visually appealing, harder to use or the update introducing in-app bugs (i.e., lagging or freezing). Previous literature focused on mental health apps also reports that issues with usability may lead to users discontinuing app usage (44). This also extends to non-mental health related apps, with surveys of app users revealing that 53% of respondents reported they would uninstall an app following severe functionality issues (80). However, regular app updates are important for increasing user engagement and may help avoid drop-out if they are of high quality (43). Therefore, app developers should aim to perform a comprehensive app testing period before deploying any app updates. Ideally, this would also include a period of A-B testing of the old vs. the new version of the app in consultation with a subset of active app users or other individuals in the population of interest, to ensure the new update is functional and acceptable.

Reviews mentioning app use discontinuation in relation to a paywall were related to the addition of a subscription fee needed to access some or all of the features offered within the app. In some cases, these paywalls were not disclosed at all within the app store descriptions before downloads, or the extent to which they would impact the users access to the app was not disclosed (i.e., the user could access all features but one). Previous findings revealed that these hidden costs were frustrating to users (53) and even indicate that a paywall may encourage users to search for other apps which offer the same features at a lower cost or no cost (55). Interestingly, when exploring themes that co-occurred with app use discontinuation, accessibility (paywall) and functionality (bad update) in combination were more common than either theme alone. Hence, in our dataset, functionality issues alone were not the most common reason for app use discontinuation despite previous literature indicating this is a key factor. Additionally, this finding suggests that paywalls are considered more of a barrier to sustained app use when introduced as part of an update than if present from the initial download. Users may view a paywall introduced in an app update as unexpected and a hidden app cost: known to be a barrier to health app usage (81). In light of these findings, app developers should notify app users if and when an app update will also include the introduction of a paywall. This, along with an explanation of why the paywall introduction is required, may promote users' acceptance of the update and prevent use discontinuation. Additionally, app developers should consider, where practical, to allow users continued access to a scaled back version of the app for free following an update.

### Recommendations

- App developers should aim to expand the analysis of user feedback to incorporate written reviews alongside star ratings in order to obtain a more comprehensive picture of user perspectives to increase engagement and avoid app use discontinuation.
- App developers should consider additional methods to collect user feedback data in order to capture the full spectrum of user experiences, including those of users who may not leave an app store review.
- Users seem to value promoting understanding of or responsibility for mental health when using apps which include a mental health screening and/or diagnostic assessment. With this in mind, providing an accurate picture of user's mental health is of paramount importance. Therefore, we recommend that app developer implement high quality, validated assessments within mental health apps to ensure a high level of screening and/or diagnostic accuracy.
- Before implementation of a new update, extensive testing should be performed to ensure the quality of the app is still high and its functionality is intact. Additionally, if a paywall is to be introduced, work should be undertaken to ensure users do not feel blindsided by the introduction of in-app purchases; or, an old or basic version of the app should remain available for those users who do not wish to pay to access the app.
- If possible, app developers should consider adapting the core screening and/or diagnostic assessment into a longitudinal tracking tool in an effort to increase user engagement as well as increased mental health understanding and/or responsibility.

### Limitations

A limitation of the current study is related to performing searches in app stores. App stores are not designed for robust, rigorous searches unlike electronic journal databases (58). Searching in app stores may introduce potential challenges to reproduce any findings (58). In an effort to address this, the PRISMA-ScR checklist was used to improve reporting of the methods implemented and searches which were conducted (see Supplementary Material 1) (58, 59).

Additionally, whilst we employed a thematic analysis in the current study for identifying theme frequencies and allowing for theme comparisons, we recognize that the results of any analysis may have been influenced by possible reviewer bias. We attempted to minimize any bias during the qualitative analysis by performing a dual independent review process at each stage of the analysis.

Furthermore, due to the feasibility constraints imposed by performing a manual analysis with multiple independent reviewers, only a subset of all app store reviews were analyzed. However, by only considering the “most helpful” reviews (i.e., upvoted by users for their helpfulness) as per previous similar literature (54) and filtering reviews from the last 6 months, we hope to have compiled a dataset that depicts an up-to-date and relevant picture of app users' perspectives.

Other limitations are beyond the control of the authors, for instance the results from app stores searches are based on factors beyond search terms, such as whether the app offers in-app purchases and the number of downloads (64). There are also limitations related to the accuracy of the data, as the descriptive app information is directly provided by the app developers (82) without any information of accuracy checks being performed by the app stores. Furthermore, app developers are able to vary the information provided in the app store descriptions (i.e., in-app screenshots, app keyword, app description) based on geographical locations (83, 84).

## Conclusions

To conclude, app reviews remain a valuable yet underutilized resource which offer an abundance of insights and actionable information provided directly by patients and users. Considering information provided within the user reviews can inform app design, and ensure the app is suitable for purpose as determined by end users themselves. Overall, apps which include a mental health screening and/or diagnostic assessment are perceived positively, with very few users reporting app use discontinuation and many users valuing an apps' ability to increase understanding of their mental health. However, there are clear areas of improvement which can be considered by app development teams to avoid negative user experiences and app use discontinuation. These include avoiding the implementation of an unexpected paywall and extensive app testing before an update is released. In addition, consideration of the quality of assessments delivered *via* mental health apps should be undertaken. Principally, ensuring that the included assessment is high-quality, validated, and confers a high degree of accuracy. In doing so, developers will contribute to an increased likelihood that the app will provide an accurate picture of the user's mental health, which was identified as the most commonly cited indicator of app performance rating by users.

## Data Availability Statement

The original contributions presented in the study are included in the article/Supplementary Material, further inquiries can be directed to the corresponding author.

## Author Contributions

EF and NM-K conceived the study focus. The searches of the app stores and inclusion decisions were made by EF, BS, and NM-K. Reviews from the app stores were scraped by EF and TM. Code was developed for scraping of the Apple app store by TM. The qualitative analysis was performed by EF as first reviewer and BS and NM-K as the second reviewers. EF prepared the manuscript with revisions from BS, NM-K, TM, and SB. All authors contributed to the article and approved the submitted version.

## Funding

This study was funded by the Stanley Medical Research Institute (grant 07R-1888).

## Conflict of Interest

SB is a director of Psynova Neurotech Ltd and Psyomics Ltd. SB has financial interests in Psyomics Ltd. The remaining authors declare that the research was conducted in the absence of any commercial or financial relationships that could be construed as a potential conflict of interest.

## Publisher's Note

All claims expressed in this article are solely those of the authors and do not necessarily represent those of their affiliated organizations, or those of the publisher, the editors and the reviewers. Any product that may be evaluated in this article, or claim that may be made by its manufacturer, is not guaranteed or endorsed by the publisher.

## References

[1] Office for National Statistics. Coronavirus and depression in adults, Great Britain: July to August 2021 (2021). Available online at: https://www.ons.gov.uk/peoplepopulationandcommunity/wellbeing/articles/coronavirusanddepressioninadultsgreatbritain/julytoaugust2021 (accessed December 1, 2021).

[2] Armitage R. Antidepressants, primary care, and adult mental health services in England during COVID-19. Lancet Psychiatry. (2021) 8:e3. 10.1016/S2215-0366(20)30530-7 33485423 PMC9764342

[3] Ettman CK Abdalla SM Cohen GH Sampson L Vivier PM Galea S. Prevalence of depression symptoms in US adults before and during the COVID-19 pandemic. JAMA Netw Open. (2020) 3:e2019686. 10.1001/jamanetworkopen.2020.19686 32876685 PMC7489837

[4] Xiong J Lipsitz O Nasri F Lui LMW Gill H Phan L. Impact of COVID-19 pandemic on mental health in the general population: a systematic review. J Affect Disord. (2020) 277:55–64. 10.1016/j.jad.2020.08.001 32799105 PMC7413844

[5] Office for National Statistics. Are we facing a mental health pandemic? (2021). Available online at: https://blog.ons.gov.uk/2021/05/05/are-we-facing-a-mental-health-pandemic/ (accessed December 3, 2021).

[6] Nuffield Trust. What impact had Covid-19 had on mental health services? (2020). Available online at: https://www.nuffieldtrust.org.uk/news-item/what-impact-has-covid-19-had-on-mental-health-services (accessed December 3, 2021).

[7] British Medical Association. Measuring progress: Commitments to support and expand the mental health workforce in England (2019). Available online at: https://www.bma.org.uk/media/2405/bma-measuring-progress-of-commitments-for-mental-health-workforce-jan-2020.pdf (accessed August 18, 2021).

[8] British Medical Association. The impact of COVID-19 on mental health in England; Supporting services to go beyond parity of esteem (2020). Available online at: https://www.bma.org.uk/media/2750/bma-the-impact-of-covid-19-on-mental-health-in-england.pdf (accessed August 18, 2021).

[9] Abbas MJ Kronenberg G McBride M Chari D Alam F Mukaetova-Ladinska E. The early impact of the COVID-19 pandemic on acute care mental health services. Psychiatr Serv. (2021) 72:242–6. 10.1176/appi.ps.202000467 33076794

[10] Reichert A Jacobs R. The impact of waiting time on patient outcomes: Evidence from early intervention in psychosis services in England. Health Econ. (2018) 27:1772–87. 10.1002/hec.3800 30014544 PMC6221005

[11] Goldberg JF Ernst CL. Features associated with the delayed initiation of mood stabilizers at illness onset in bipolar disorder. J Clin Psychiatry. (2002) 63:985–91. 10.4088/JCP.v63n1105 12444811

[12] Altamura AC Dell'Osso B Berlin HA Buoli M Bassetti R Mundo E. Duration of untreated illness and suicide in bipolar disorder: a naturalistic study. Eur Arch Psychiatry Clin Neurosci. (2010) 260:385–91. 10.1007/s00406-009-0085-2 19911248

[13] Post RM Leverich GS Kupka RW Keck PE McElroy SL Altshuler LL. Early-onset bipolar disorder and treatment delay are risk factors for poor outcome in adulthood. J Clin Psychiatry. (2010) 71:864–72. 10.4088/JCP.08m04994yel 20667291

[14] Altamura AC Dell'Osso B Mundo E Dell'Osso L. Duration of untreated illness in major depressive disorder: a naturalistic study. Int J Clin Pract. (2007) 61:1697–700. 10.1111/j.1742-1241.2007.01450.x 17877655

[15] Albert U Barbaro F Bramante S Rosso G De Ronchi D Maina G. Duration of untreated illness and response to SRI treatment in obsessive-compulsive disorder. Eur Psychiatry. (2019) 58:19–26. 10.1016/j.eurpsy.2019.01.017 30763828

[16] Altamura AC Dell'Osso B Vismara S Mundo E. May duration of untreated illness influence the long-term course of major depressive disorder? Eur Psychiatry. (2008) 23:92–6. 10.1016/j.eurpsy.2007.11.004 18248964

[17] Patel R Shetty H Jackson R Broadbent M Stewart R Boydell J. Delays before diagnosis and initiation of treatment in patients presenting to mental health services with bipolar disorder. PLoS ONE. (2015) 10:e0126530. 10.1371/journal.pone.0126530 25992560 PMC4439113

[18] McGorry PD Ratheesh A O'Donoghue B. Early intervention—an implementation challenge for 21st century mental health care. JAMA Psychiatry. (2018) 75:545. 10.1001/jamapsychiatry.2018.0621 29801060

[19] NHS Oxford Health. Study finds that early intervention services can save NHS millions (2016). Available online at: https://www.oxfordhealth.nhs.uk/news/study-finds-that-early-intervention-services-can-save-nhs-millions/ (accessed August 18, 2021).

[20] Correll CU Galling B Pawar A Krivko A Bonetto C Ruggeri M. Comparison of early intervention services vs treatment as usual for early-phase psychosis. JAMA Psychiatry. (2018) 75:555. 10.1001/jamapsychiatry.2018.0623 29800949 PMC6137532

[21] National Mental Health Development Unit. Factfile 3 The costs of mental ill health (2021. Available online at: https://www.networks.nhs.uk/nhs-networks/regional-mental-health-workshop-mids-east/documents/supporting-materials/nmhdu-factfile-3.pdf (accessed August 18, 2021).

[22] Cano A Sprafkin RP Scaturo DJ Lantinga LJ Fiese BH Brand F. Mental health screening in primary care. Prim Care Companion J Clin Psychiatry. (2001) 03:206–10. 10.4088/PCC.v03n0503 15014574 PMC181216

[23] Sin J Galeazzi G McGregor E Collom J Taylor A Barrett B. Digital interventions for screening and treating common mental disorders or symptoms of common mental illness in adults: systematic review and meta-analysis. J Med Internet Res. (2020) 22:e20581. 10.2196/20581 32876577 PMC7495259

[24] Spadaro B Martin-Key NA Bahn S. Building the digital mental health ecosystem: opportunities and challenges for mobile health innovators. J Med Internet Res. (2021) 23:e27507. 10.2196/27507 34643537 PMC8552100

[25] Mind. 40 per cent of all GP appointments about mental health (2018). Available online at: https://www.mind.org.uk/news-campaigns/news/40-per-cent-of-all-gp-appointments-about-mental-health/ (accessed December 3, 2021).

[26] NHS. Active signposting frees up 80 inappropriate GP appointments a week. Available online at: https://www.england.nhs.uk/gp/case-studies/active-signposting-frees-up-80-inappropriate-gp-appointments-a-week/ (accessed January 4, 2022).

[27] Schmitz N Kruse J Tress W. Improving screening for mental disorders in the primary care setting by combining the GHQ-12 and SCL-90-R subscales. Compr Psychiatry. (2001) 42:166–73. 10.1053/comp.2001.19751 11244154

[28] Bucci S Schwannauer M Berry N. The digital revolution and its impact on mental health care. Psychol Psychother Theory, Res Pract. (2019) 92:277–97. 10.1111/papt.12222 30924316

[29] Torous J Haim A. Dichotomies in the development and implementation of digital mental health tools. Psychiatr Serv. (2018) 69:1204–6. 10.1176/appi.ps.201800193 30256182 PMC6408291

[30] Schueller SM Hunter JF Figueroa C Aguilera A. Use of digital mental health for marginalized and underserved populations. Curr Treat Options Psychiatry. (2019) 6:243–55. 10.1007/s40501-019-00181-z

[31] GOV.UK. Policy paper COVID-19 mental health and wellbeing recovery action plan (2021). Available online at: https://www.gov.uk/government/publications/covid-19-mental-health-and-wellbeing-recovery-action-plan/covid-19-mental-health-and-wellbeing-recovery-action-plan (accessed December 3, 2021).

[32] Naslund JA Gonsalves PP Gruebner O Pendse SR Smith SL Sharma A. Digital innovations for global mental health: opportunities for data science, task sharing, and early intervention. Curr Treat Options Psychiatry. (2019) 6:337–51. 10.1007/s40501-019-00186-8 32457823 PMC7250369

[33] Torous J Roberts LW. Needed innovation in digital health and smartphone applications for mental health transparency and trust. JAMA Psychiatry. (2017) 74:437–8. 10.1001/jamapsychiatry.2017.0262 28384700

[34] ORCHA. COVID-19: digital health trends & opportunities for 2021 (2021). Available online at: https://orchahealth.com/wp-content/uploads/2021/01/COVID_Report_Jan_2021_final-version.pdf (accessed August 20, 2021).

[35] Chan AHY Honey MLL. User perceptions of mobile digital apps for mental health: acceptability and usability-an integrative review. J Psychiatr Ment Health Nurs. (2022) 29:147–68. 10.1111/jpm.12744 33604946

[36] Naslund JA Marsch LA McHugo GJ Bartels SJ. Emerging mHealth and eHealth interventions for serious mental illness: a review of the literature. J Ment Heal. (2015) 24:321–32. 10.3109/09638237.2015.1019054 26017625 PMC4924808

[37] BinDhim NF Shaman AM Trevena L Basyouni MH Pont LG Alhawassi TM. Depression screening *via* a smartphone app: cross-country user characteristics and feasibility. J Am Med Informatics Assoc. (2015) 22:29–34. 10.1136/amiajnl-2014-002840 25326599 PMC4433364

[38] BinDhim NF Alanazi EM Aljadhey H Basyouni MH Kowalski SR Pont LG. Does a mobile phone depression-screening app motivate mobile phone users with high depressive symptoms to seek a health care professional's help? J Med Internet Res. (2016) 18:e156. 10.2196/jmir.5726 27349441 PMC4962995

[39] Torous J Nicholas J Larsen ME Firth J Christensen H. Clinical review of user engagement with mental health smartphone apps: evidence, theory and improvements. Evid Based Ment Heal. (2018) 21:116–9. 10.1136/eb-2018-102891 29871870 PMC10270395

[40] Fleming T Bavin L Lucassen M Stasiak K Hopkins S Merry S. Beyond the trial: systematic review of real-world uptake and engagement with digital self-help interventions for depression, low mood, or anxiety. J Med Internet Res. (2018) 20:e199. 10.2196/jmir.9275 29875089 PMC6010835

[41] Torous J Lipschitz J Ng M Firth J. Dropout rates in clinical trials of smartphone apps for depressive symptoms: a systematic review and meta-analysis. J Affect Disord. (2020) 263:413–9. 10.1016/j.jad.2019.11.167 31969272

[42] Meyerowitz-Katz G Ravi S Arnolda L Feng X Maberly G Astell-Burt T. Rates of attrition and dropout in app-based interventions for chronic disease: systematic review and meta-analysis. J Med Internet Res. (2020) 22:e20283. 10.2196/20283 32990635 PMC7556375

[43] Alqahtani F Orji R. Usability issues in mental health applications. Adjunct Publication of the 27th Conference on User Modeling, Adaptation and Personalization. New York, NY, USA: ACM (2019). p. 343–8. 10.1145/3314183.3323676

[44] Alqahtani F Orji R. Insights from user reviews to improve mental health apps. Health Informatics J. (2020) 26:2042–66. 10.1177/1460458219896492 31920160

[45] Borghouts J Eikey E Mark G De Leon C Schueller SM Schneider M. Barriers to and facilitators of user engagement with digital mental health interventions: systematic review. J Med Internet Res. (2021) 23:e24387. 10.2196/24387 33759801 PMC8074985

[46] ORCHA. What is the Health of Mental Health Apps? (2020). Available online at: https://orchahealth.com/health-of-mental-health-apps/ (accessed December 6, 2021).

[47] Iacob C Harrison R. Retrieving and analyzing mobile apps feature requests from online reviews. 2013 10th Working Conference on Mining Software Repositories (MSR). San Francisco, CA: IEEE (2013). p. 41–4. 10.1109/MSR.2013.6624001

[48] Iacob C Veerappa V Harrison R. What are you complaining about?: a study of online reviews of mobile applications. In: Proceedings of the 27th International BCS Human Computer Interaction Conference (London). (2013). 10.14236/ewic/HCI2013.38

[49] Apptentive. Mobile App Ratings and Reviews: Where to Start and How to Win (2021). Available online at: https://go.apptentive.com/rs/170-TZF-108/images/Ratings-Reviews-1.pdf?mkt_tok=MTcwLVRaRi0xM DgAAAF-bWzonyVyyjyamC8dcN4qIcF5cja-_kc-1qaVfvX67Khl1kvIsNiattXD2udRHQYw1SubrrqHWt0U5GpG-Sb5RypGFT3ALcRTThaxlOMWfQ (accessed August 20, 2021).

[50] Al-Natour S Turetken O. A comparative assessment of sentiment analysis and star ratings for consumer reviews. Int J Inf Manage. (2020) 54:102132. 10.1016/j.ijinfomgt.2020.102132

[51] Stawarz K Preist C Tallon D Wiles N Coyle D. User experience of cognitive behavioral therapy apps for depression: an analysis of app functionality and user reviews. J Med Internet Res. (2018) 20:e10120. 10.2196/10120 29875087 PMC6010839

[52] Nicholas J Fogarty AS Boydell K Christensen H. The reviews are in: a qualitative content analysis of consumer perspectives on apps for bipolar disorder. J Med Internet Res. (2017) 19:e105. 10.2196/jmir.7273 28389420 PMC5400886

[53] Widnall E Grant CE Wang T Cross L Velupillai S Roberts A. User perspectives of mood-monitoring apps available to young people: qualitative content analysis. JMIR mHealth uHealth. (2020) 8:e18140. 10.2196/18140 33037875 PMC7585773

[54] Stawarz K Cox AL Blandford A. Don't forget your pill! In: Proceedings of the SIGCHI Conference on Human Factors in Computing Systems. New York, NY, USA: ACM (2014). p. 2269–78. 10.1145/2556288.2557079

[55] Oyebode O Alqahtani F Orji R. Using machine learning and thematic analysis methods to evaluate mental health apps based on user reviews. IEEE Access. (2020) 8:111141–58. 10.1109/ACCESS.2020.3002176

[56] Pang B Lee L. Opinion mining and sentiment analysis. Found Trends® Inf Retr. (2008) 2:1–135. 10.1561/1500000011

[57] Braun V Clarke V. Using thematic analysis in psychology. Qual Res Psychol. (2006) 3:77–101. 10.1191/1478088706qp063oa

[58] Grainger R Devan H Sangelaji B Hay-Smith J. Issues in reporting of systematic review methods in health app-focused reviews: a scoping review. Health Informatics J. (2020) 26:2930–45. 10.1177/1460458220952917 32914696

[59] Tricco AC Lillie E Zarin W O'Brien KK Colquhoun H Levac D. PRISMA extension for scoping reviews (PRISMA-ScR): checklist and explanation. Ann Intern Med. (2018) 169:467–73. 10.7326/M18-0850 30178033

[60] Funnell E. User reviews of mental health screening and diagnostic apps: a sentiment and thematic analysis (OSF registered study plan). (2021). 10.17605/OSF.IO/DZBPJ

[61] M-Health Index and Navigation Database. Framework (2020). Available online at: https://mindapps.org/ (accessed August 11, 2021).

[62] Colaboratory. Create a Dataset for Sentiment Analysis. Available online at: https://colab.research.google.com/github/curiousily/Getting-Things-Done-with-Pytorch/blob/master/07.create-dataset-for-sentiment-analysis.ipynb (accessed August 18, 2021).

[63] npm Inc. app-store-scraper (2021). Available online at: https://www.npmjs.com/package/app-store-scraper (accessed September 4, 2021).

[64] Store Maven. Ranking Factors for App Store and Google Play (2020). Available online at: https://www.storemaven.com/academy/app-store-and-google-play-algorithms/ (accessed February 18, 2022).

[65] App Radar. What is App Store Optimization: Ultimate Guide to ASO in 2022 (2022). Available online at: https://appradar.com/academy/what-is-app-store-optimization-aso (accessed February 24, 2022).

[66] Shen N Levitan M-J Johnson A Bender JL Hamilton-Page M Jadad A. Finding a depression app: a review and content analysis of the depression app marketplace. JMIR mHealth uHealth. (2015) 3:e16. 10.2196/mhealth.3713 25689790 PMC4376135

[67] Larsen ME Nicholas J Christensen H. Quantifying app store dynamics: longitudinal tracking of mental health apps. JMIR mHealth uHealth. (2016) 4:e96. 10.2196/mhealth.6020 27507641 PMC4995352

[68] Apptentive. Mobile App Ratings and Reviews Aren't Enough–Here's Why (2022). Available online at: https://www.apptentive.com/blog/2022/01/27/mobile-app-ratings-and-reviews-arent-enough-heres-why/ (accessed February 24, 2022).

[69] Minen MT Gopal A Sahyoun G Stieglitz E Torous J. The functionality, evidence, and privacy issues around smartphone apps for the top neuropsychiatric conditions. J Neuropsychiatry Clin Neurosci. (2021) 33:72–9. 10.1176/appi.neuropsych.19120353 32669020 PMC8670295

[70] Alshayban A Ahmed I Malek S. Accessibility issues in Android apps. In: Proceedings of the ACM/IEEE 42nd International Conference on Software Engineering. New York, NY, USA: ACM (2020). p. 1323–34. 10.1145/3377811.3380392

[71] Adhikari R Richards D Scott K. Security and privacy issues related to the use of mobile health apps. In: Proceedings of the 25th Australasian Conference on Information Systems, 8th-10th December. Auckland, New Zealand: ACIS (2014).

[72] Minen MT Stieglitz EJ Sciortino R Torous J. Privacy issues in smartphone applications: an analysis of headache/migraine applications. Headache J Head Face Pain. (2018) 58:1014–27. 10.1111/head.13341 29974470 PMC6347475

[73] Scoccia GL Peruma A Pujols V Malavolta I Krutz DE. Permission issues in open-source android apps: an exploratory study. In: 2019 19th International Working Conference on Source Code Analysis and Manipulation (SCAM). Cleveland, OH: IEEE (2019). p. 238–49. 10.1109/SCAM.2019.00034

[74] Mirea D-M Martin-Key NA Barton-Owen G Olmert T Cooper JD Han SYS. Impact of a web-based psychiatric assessment on the mental health and well-being of individuals presenting with depressive symptoms: longitudinal observational study. JMIR Ment Heal. (2021) 8:e23813. 10.2196/23813 33616546 PMC7939939

[75] Martin-Key NA Spadaro B Funnell E Barker EJ Schei TS Tomasik J. The current state and validity of digital assessment tools for psychiatry: systematic review. JMIR Ment Heal. (2022) 9:e32824. 10.2196/32824 35353053 PMC9008525

[76] Nayebi M Adams B Ruhe G. Release Practices for Mobile Apps–What do Users and Developers Think? In: 2016 IEEE 23rd International Conference on Software Analysis, Evolution, and Reengineering (SANER). Osaka: IEEE (2016). p. 552–62. 10.1109/SANER.2016.116

[77] Thach KS. User's perception on mental health applications: a qualitative analysis of user reviews. In: 2018 5th NAFOSTED Conference on Information and Computer Science (NICS). Ho Chi Minh City: IEEE (2018). p. 47–52. 10.1109/NICS.2018.8606901

[78] Kelley C Lee B Wilcox L. Self-tracking for Mental Wellness. In: Proceedings of the 2017 CHI Conference on Human Factors in Computing Systems. New York, NY, USA: ACM (2017). p. 629–41. 10.1145/3025453.3025750 PMC5600512 28920106

[79] Schueller SM Neary M Lai J Epstein DA. Understanding people's use of and perspectives on mood-tracking apps: interview study. JMIR Ment Heal. (2021) 8:e29368. 10.2196/29368 34383678 PMC8387890

[80] RAYGUN. Mobile application abandonment: common reasons (2016). Available online at: https://raygun.com/blog/mobile-application-abandonment/ (accessed February 24, 2022).

[81] Krebs P Duncan DT. Health app use among US mobile phone owners: a national survey. JMIR mHealth uHealth. (2015) 3:e101. 10.2196/mhealth.4924 26537656 PMC4704953

[82] Paglialonga A Lugo A Santoro E. An overview on the emerging area of identification, characterization, and assessment of health apps. J Biomed Inform. (2018) 83:97–102. 10.1016/j.jbi.2018.05.017 29852318

[83] Grundy QH Wang Z Bero LA. Challenges in assessing mobile health app quality: a systematic review of prevalent and innovative methods. Am J Prev Med. (2016) 51:1051–9. 10.1016/j.amepre.2016.07.009 27659122

[84] App Store Connect Help. Required, localizable, and editable properties (2022). Available online at: https://help.apple.com/app-store-connect/#/devfc3066644 (accessed February 18, 2022).

